# Impaired fasting glucose as an independent risk factor for hypertension among healthy middle-aged Japanese subjects with optimal blood pressure: the Yuport Medical Checkup Centre retrospective cohort study

**DOI:** 10.1186/1758-5996-5-81

**Published:** 2013-12-20

**Authors:** Masaaki Morio, Machiko Inoue, Kazuo Inoue, Kimihiko Akimoto

**Affiliations:** 1 Department of Family Medicine, Kochi Medical School, Kohasu, Oko-cho, Nankoku, Kochi, Japan; 2 Department of Community Medicine, Teikyo University School of Medicine, 2-11-1 Kaga, Itabashi-ku, Tokyo, Japan; 3 Department of Community Medicine, Chiba Medical Center, Teikyo University School of Medicine, 3426-3, Anesaki, Ichihara, Chiba, Japan; 4 Akimoto Occupational Health Consultant Office, 4-7-22 Kudan-minami, Chiyoda-ku, Tokyo, Japan

**Keywords:** Fasting plasma glucose, Impaired fasting glucose, Optimal blood pressure, Hypertension

## Abstract

**Background:**

This study aimed at investigating whether impaired fasting glucose (IFG) is an independent risk factor for incident hypertension among middle-aged Japanese subjects with optimal blood pressure (OBP).

**Findings:**

This retrospective cohort study was conducted in 2943 non-diabetic and non-hypertensive subjects aged 40–64 years, who participated in a voluntary health check-up program during the baseline (1998–2002) and follow-up periods (2002–2006). A multiple logistic regression model was utilized to calculate the odds ratio (OR) of incident hypertension among men and women with IFG and OBP. OBP was defined as systolic blood pressure (SBP) <120 mmHg and diastolic blood pressure (DBP) <80 mmHg, with no known history of hypertension. In this study, hypertension was defined as SBP ≥140 mmHg and DBP ≥90 mmHg or by a self-reported clinical diagnosis of hypertension. After the mean follow-up period of 5.6 years, the incidence of hypertension in men and women was 5.7% (73/1270) and 3.8% (62/1673), respectively. The age-adjusted ORs for incident hypertension in men and women with IFG were 1.95 (95% CI, 1.21–3.15) and 3.54 (95% CI, 2.00–6.27), respectively. After adjusting for age, systolic blood pressure, body mass index, total cholesterol, triglyceride, high-density lipoprotein cholesterol, and uric acid, the ORs for hypertension were 1.66 (95% CI; 1.02–2.70) for men and 2.62 (95% CI, 1.45–4.73) for women.

**Conclusion:**

The study results show that IFG may act as an independent risk factor for developing hypertension in individuals with OBP.

## Background

Hypertension and diabetes are the leading morbidities affecting the general population, often concurrently, as there is substantial overlap between hypertension and diabetes in both etiology and disease mechanism [1-3]. The association between elevated blood pressure and dysglycemia has also recently been of interest [4].

In 1997, the American Diabetes Association (ADA) introduced the term ‘impaired fasting glucose’ (IFG), defined as a fasting plasma glucose (FPG) level of 110–125 mg/dL (IFG^1997^), to describe a prediabetic state of dysglycemia [5]. Accordingly, researchers began to examine whether IFG may be an independent risk factor for the development of hypertension as well. However, previous findings were inconsistent [6-10]. Although IFG and elevated FPG emerged as independent risk factors for the development of hypertension in 3 prospective studies [6-8], this relationship was not found in 2 other studies after adjusting for body mass index (BMI) or waist circumference [9,10].

Central obesity is a well-known risk factor for both diabetes and hypertension [3,11], and is thought to be a common pathway whereby obesity affects both diseases [1,2]. Simone et al. reported that abdominal obesity was a major predictor of the development of hypertension even at optimal blood pressure (OBP) [8], which suggests that atherogenic risk factors could play a major role in atherosclerosis even when blood pressure is low or at optimal levels.

The association of IFG and hypertension in those with OBP has not been investigated thoroughly. Furthermore, although the ADA lowered the FPG concentration range for diagnosis of IFG from 110–125 mg/dL to 100–125 mg/dL in 2003 [9], there have been no studies conducted to determine whether this new criterion for IFG is predictive of the development of hypertension.

In this study, we investigated whether the new criterion for IFG could be considered as an independent risk factor for incident hypertension among healthy middle-aged Japanese subjects with OBP.

## Methods

### Study subjects

We used a health screening program cohort of 13,734 healthy middle-aged Japanese subjects, who received routine medical check-ups during the baseline period between April 1998 and March 2002 at the Yuport Medical Checkup Centre in Tokyo, which has been described previously [12-15]. If subjects received more than 1 check-up during the baseline period, the initial check-up data were used. The 4-year follow-up period ended between April 2002 and March 2006, and the 6,290 subjects who did not have follow-up data were excluded. If subjects received more than 1 check-up during the follow-up period, all data were used to identify incident hypertension. This study excluded 424 subjects who had a baseline FPG ≥126 mg/dL, HbA1c ≥6.5%, or self-reported clinically diagnosed diabetes, as well as 4067 subjects with a baseline SBP ≥120 mmHg, DBP ≥80 mmHg, or self-reported clinical diagnosis of hypertension. The remaining 2943 non-diabetic and non-hypertensive patients were included as study subjects (mean follow-up, 5.6 ± 1.6 years).

### Clinical and laboratory procedures

All medical check-ups followed the same procedures during both the baseline and follow-up periods. Resting blood pressure was measured twice by well-trained nurses using a mercury column sphygmomanometer, after each subject sat quietly for at least 5 min. The average of the 2 measurements was used for analysis.

All blood samples were collected after an overnight fast. Blood samples were measured on the same autoanalyzer in the laboratory at the medical center. Plasma glucose levels were measured using the hexokinase-G6PD method (Denka Seiken, Niigata, Japan) with an interassay coefficient of variation (CV) ≤3.0%. Hemoglobin A1c (HbA1c) levels were measured using a latex immunoagglutination assay method (Determiner HbA1c, Kyowa Medex, Tokyo, Japan) with an interassay CV of 1.7–2.1%, which was aligned with the units used by the Japan Diabetes Society (JDS). The JDS value for HbA1c was converted into National Glycohemoglobin Standardization Program (NGSP) units in this study, which was calculated by the formula: HbA1c (NGSP) = HbA1c (JDS) + 0.4 [14]. Other blood tests, total cholesterol (TC), high-density lipoprotein cholesterol (HDL-C), triglycerides (TG), creatinine (Cr), and uric acid (UA), were measured using enzymatic methods (reagents supplied by Daiichi Pure Chemicals, Tokyo, Japan). Low-density lipoprotein cholesterol (LDL-C) was calculated by the Friedewald equation [16].

In accordance with the Private Information Protection Law, information that might identify subjects was kept confidential. This study was approved by the review board of the Yuport Medical Checkup Centre, and informed consent for anonymous participation in this study was obtained from each subject at every check-up.

### Diagnosis of hypertension and IFG

The Seventh Report of the Joint National Committee on Prevention, Detection, Evaluation, and Treatment of High Blood Pressure (JNC 7) criteria were utilized [17] to define OBP as SBP <120 mmHg and DBP <80 mmHg. Hypertension was defined as SBP ≥140 mmHg and DBP ≥90 mmHg, or as self-reported clinically diagnosed hypertension. FPG categories were defined by using the 2003 ADA criteria [18] as normal fasting glucose (NFG); FPG ≤99 mg/dL and IFG; FPG of 100–125 mg/dL.

### Statistical analysis

Baseline characteristics for all subjects are presented as mean ± standard deviation (SD). A Students t-test or chi-square test was used to test for differences in mean values and frequencies between men and women. We calculated the odds ratios (ORs) and 95% confidence intervals (CIs) to assess the association between IFG (versus NFG) at baseline and the incidence of hypertension at follow-up using multiple logistic regression analyses. We used two multivariable models: model 1, adjusted for age; and model 2, adjusted for age, BMI, SBP, TC, TG, HDL-C and UA. All reported *P*-values were two-tailed, and *P* < 0.05 was considered statistically significant. All statistical analyses were performed using SPSS version 19 (SPSS Inc., Japan).

## Results

The baseline characteristics of subjects and the development of hypertension by sex are given in Table 1. Women were older and had significantly lower SBP, DBP, TG, Cr, and UA, while they had higher TC, HDL-C, and LDL-C than men. The HbA1c level was not statistically different between the sexes. The incidence of hypertension at the mean follow-up time of 5.6 years was 5.7% and 3.7% in men and women, respectively.

**Table 1:** Baseline characteristics and incidence of hypertension

	**Total**	**Men**	**Women**	
	**N = 2943**	**N = 1270 (43.2%)**	**N = 1673 (56.8%)**	*P*-value^*^
Age (years)	51.7 ± 6.7	51.0 ± 6.7	52.2 ± 6.7	<0.001
BMI (kg/m^2^)	22.1 ± 2.6	22.7 ± 2.6	21.5 ± 2.6	<0.001
SBP (mmHg)	107.5 ± 7.9	108.8 ± 7.2	106.5 ± 8.3	<0.001
DBP (mmHg)	65.8 ± 6.2	67.5 ± 5.6	64.5 ± 6.4	<0.001
FPG (mg/dL)	92.8 ± 8.5	96.1 ± 8.5	90.4 ± 7.5	<0.001
HbA1c (%)	5.4 ± 0.4	5.4 ± 0.4	5.4 ± 0.4	0.517
TC (mg/dL)	201.6 ± 33.7	194.8 ± 32.5	206.7 ± 33.7	<0.001
TG (mg/dL)	102.6 ± 62.0	124.8 ± 76.3	85.7 ± 40.9	<0.001
HDL-C (mg/dL)	60.3 ± 15.6	52.7 ± 13.3	66.0 ± 14.7	<0.001
LDL-C (mg/dL)	120.8 ± 30.1	117.1 ± 30.2	123.6 ± 30.8	<0.001
Cr (mg/dL)	0.70 ± 0.15	0.82 ± 0.13	0.61 ± 0.09	<0.001
UA (mg/dL)	5.0 ± 1.3	5.9 ± 1.2	4.4 ± 0.9	<0.001
Follow-up (years)	5.6 ± 1.6	5.7 ± 1.5	5.5 ± 1.6	<0.001
HT (%)	135 (4.6%)	73 (5.7%)	62 (3.7%)	0.010

Data are presented as mean ± SD or number (%).

^*^Mean or frequency (%) difference between men and women.

BMI, body mass index; SBP, systolic blood pressure; DBP, diastolic blood pressure; FPG, fasting plasma glucose; HbA1c, glycosylated hemoglobin; TC, total cholesterol; TG, triglyceride; HDL-C, high density lipoprotein cholesterol; LDL-C, low density lipoprotein cholesterol; Cr, creatinine; UA, uric acid; HT, hypertension.

The incidence of hypertension in the FPG categories and the multivariate-adjusted ORs for developing hypertension in subjects with OBP and IFG when compared with NFG by sex are given in Table 2. The age-adjusted OR (shown as Model 1) for the development of hypertension was 1.95 (95% CI, 1.21–3.15) and 3.54 (95% CI, 2.00–6.27) for men and women, respectively. After adjusting for age, BMI, SBP, TC, TG, HDL-C and UA, the OR (shown as Model 2) for hypertension was 1.66 (95% CI, 1.02–2.70) and 2.62 (95% CI, 1.45–4.73) for men and women, respectively. Age and SBP in both sexes and BMI in women also remained as predictors of incident hypertension. The IFG in both sexes was significantly and positively associated with the risk of hypertension.

**Table 2:** Odds ratio for incident hypertension in middle-aged Japanese subjects with OBP by sex

	**FPG categories at baseline**	**Men**		**Women**	
		**Incidence of hypertension, (%)**		**Incidence of hypertension, (%)**	
	**NFG**	**40/848 (4.5%)**		**43/1453 (2.9%)**	
	**IFG**	**33/349 (8.6%)**		**19/158 (10.7%)**	
		**OR (95% CI)**	***P-*** **value**	**OR (95% CI)**	***P-*** **value**
Model 1^*^	IFG vs. NFG	1.95 (1.21-3.15)	0.006	3.54 (2.00-6.27)	<0.001
Model 2^**^	IFG vs. NFG	1.66 (1.02-2.70)	0.043	2.62 (1.45-4.73)	0.001
	Age (years)	1.04 (1.00-1.08)	0.046	1.05 (1.00-1.10)	0.042
	BMI (kg/m^2^)	1.06 (0.95-1.17)	0.332	1.17 (1.06-1.29)	0.002
	SBP (mmHg)	1.12 (1.07-1.18)	<0.001	1.09 (1.05-1.14)	<0.001
	TC (per 10 mg/dL)	1.03 (0.95-1.11)	0.478	0.94 (0.86-1.04)	0.228
	TG (per 10 mg/dL)	1.03 (1.00-1.06)	0.079	1.06 (0.99-1.12)	0.079
	HDL-C (per 10 mg/dL)	1.00 (0.80-1.25)	1.000	0.96 (0.75-1.22)	0.727
	UA (mg/dL)	1.10 (0.90-1.34)	0.361	1.17 (0.89-1.55)	0.268

^*^Model 1: adjusted for age.

^**^Model 2: adjusted for age, BMI, SBP, TC, TG, HDL-C, and UA.

FPG, fasting plasma glucose; NFG, normal fasting glucose; IFG, impaired fasting glucose.

## Discussion

The main finding of this study was that IFG may act as an independent risk factor for the development of hypertension in middle-aged healthy Japanese subjects with OBP. In 1997, after the ADA introduced IFG^1997^[5], various prospective studies have demonstrated that impaired glucose tolerance (IGT), another form of dysglycemia, was an independent risk factor for incident hypertension [4,19-21]. However, the results from previous investigations of the association between IFG^1997^ or elevated FPG and the risk for hypertension are conflicting [6-10], with 3 positive and 2 negative results.

This study has some methodological strengths as compared with previous studies. First, our cohort population was relatively large and had a single homogenous ethnicity. Second, all subjects were examined at one site, and laboratory samples were measured using the same method both at baseline and follow-up.

Our study had several limitations. First, we could not include several possible confounding variables that are known hypertension risk factors, such as plasma insulin, family history, alcohol and salt consumption, smoking, waist circumference, and socioeconomic status. These variables could be relevant for explaining the relationship between IFG and the risk for hypertension. Second, incident hypertension was defined by a single basal measurement of blood pressure, which was repeated at follow-up, or by a self-reported clinical diagnosis of hypertension. Third, the study subjects were men and women who had regular check-ups at a medical center in Tokyo, and they may be a healthier subset of the general population.

In summary, this study demonstrated an association between IFG and the development of hypertension among relatively lean Japanese subjects with OBP after the ADA lowered the FPG threshold value for IFG from 110 to 100 mg/dL in 2003. The findings may help clinicians identify those who are at higher risk for hypertension.

## Competing interests

The authors declare that they have no competing interests.

## Authors’ contributions

MM, MI, KI, and KA conceived and designed the experiments. KA was responsible for data collection and performed the experiments. MM performed the statistical analysis and drafted the manuscript. MI and KI provided the critical revision. All authors have given final approval of the version to be published.
